# Evaluation of finger cartilage composition in recreational climbers with 7 Tesla T2 mapping magnetic resonance imaging

**DOI:** 10.3389/fspor.2023.1248581

**Published:** 2023-09-27

**Authors:** Thomas Bayer, Marie-Jo Brockhoff, Armin M. Nagel, Werner Adler, Christoph Lutter, Rolf Janka, Rafael Heiss, Michael Uder, Frank W. Roemer

**Affiliations:** ^1^ Department of Radiology, University Hospital Erlangen, Friedrich-Alexander-University Erlangen-Nuremberg, Germany; ^2^ Institute of Neuroradiology and Radiology, Klinikum Fürth, Fürth, Germany; ^3^ Division of Medical Physics in Radiology, German Cancer Research Centre (DKFZ), Heidelberg, Germany; ^4^ Department of Biometry and Epidemiology, University of Erlangen-Nuremberg, Erlangen, Germany; ^5^ Department of Orthopedics, University Medical Center, Rostock, Erlangen, Germany; ^6^ Boston University Chobanian & Avedisian School of Medicine, Boston, MA, United States

**Keywords:** climbing, finger, cartilage, mapping, magenetic resonance imaging

## Abstract

**Purpose:**

Sport climbing may lead to tissue adaptation including finger cartilage before apparent surface damage is detectable. The main aim was to assess finger cartilage composition with T2 mapping in young, active climbers and to compare the results to a non-climbers' collective. A secondary aim was to compare whether differences in cartilage T2 times are observed between older vs. younger volunteers.

**Methods and materials:**

7 Tesla MRI of the fingers Dig.2–4 was performed using a multi-echo spin echo sequence. Manual segmentation of 3 ROIs at the metacarpal heads, 1 ROI at the base phalanx and 1 ROI at the proximal interphalangeal joint was performed. Included were 13 volunteers without history of trauma who are regularly performing climbing activities as a recreational sport (>20 h/month). These were age-matched with 10 control subjects not performing climbing activities.

**Results:**

Mean age was 32.4 years for the climbing group and 25.8 years for the controls. Mean T2 values for the 5 different ROIs were 42.2 ± 7.8 msec for climbers and 41.4 ± 6.8 msec for non-climbers. No significant differences were observed for T2 values between both groups. However, higher age had a significant impact on T2 values for all assessed ROIs (higher age 44.2 ± 9.5, younger age 32.9 ± 5.7, *p* = 0.001).

**Discussion:**

This study evaluated the cartilage composition of young, engaged climbers with a T2 mapping MRI technique with the purpose to depict early onset joint changes. No negative impact on cartilage composition due to the sport activity was found, whereas age-related effects on the cartilage seemed to be more prominent.

## Introduction

Climbing, as a new Olympic discipline, exposes the human body to various patterns of musculoskeletal adaptation and may be associated with a specific risk for joint, muscle or cartilage injuries (1, 2). The large community of professional and recreational athletes is the reason why knowledge about this has an increasingly great medical importance (3–7). Injuries notably concern the upper extremity and finger joints due to high loads during repetitive tension and compression motion (8). This is associated with a high incidence of acute injuries, such as finger pulley rupture, which represents the most common climbing injury (9). Concerning joint tissue adaptation as a response to repetitive stress and loading, cartilage alterations and osteoarthritis (OA) development in the finger joints have been examined in several studies but an association between climbing activity and early OA development could not be shown (10–13). Young top athletes may develop tissue adaptations such as an increase in cartilage thickness and cortical thickness (13, 14) at an early stage of their career. Studies by Pastor et al. focussed on long-term climbing athletes performing at a high level and investigated a possible connection between cartilage and cortical thickness, osteophyte development and pain symptoms in follow-up studies (12, 13). Studies investigating a potential impact on finger cartilage for recreational climbing activities at an early stage are pending. However, knowledge about the earliest cartilage changes caused by climbing would be relevant, particularly in the early phase of an athlete's career. Sport behavioural adaptions with improved or gentler climbing techniques or training methods in terms of degeneration prevention might be possible.

High field magnetic resonance imaging (MRI), particularly with field strengths beyond 3 Tesla, has potential for high resolution structural imaging of the cartilage quality (15). This has been investigated in several studies, especially for bigger joints of the human skeleton, such as the hip, knee and ankle (16–18). Various MRI techniques such as delayed gadolinium-enhanced MRI (dGEMRIC) (19), diffusion weighted imaging and T2 mapping, a composite measure of water content, collagen content and organization (17), are available for this purpose. 7 Tesla MRI has the highest magnetic field strength approved for routine clinical scanning. The high magnetic field strength can be used for improvement of spatial resolution, image contrast and/or signal to noise ratios (SNR) (20). This might be beneficial for imaging of thin cartilage joint layers of smaller joints (15).

The aim of our study was to investigate finger cartilage quality as characterized by T2 mapping using 7 Tesla MRI in young climbers and to compare the results to age-matched non-climbers. An additional research question was to analyse whether age had an influence on the T2 time of cartilage.

## Materials and methods

### Study population

The study included 23 healthy volunteers (22.6–47.8 y, mean age 30.5 y, m:f = 13:10), between June and October 2017. Thirteen participants were climbers (22.6–47.8 y, mean age 32.4 y, m:f = 7:6) and ten were non climbers (24.4–35.4 y, mean age 25.8 y, m:f = 6:4). The mean climbing level was 20 (min 16 max 25; on the IRCRA climbing scale ranging from 1 to 32), the mean climbing career duration was 7.7 years (min 5.9 max 18.9 years), the average regular climbing time was 20.9 h/month. This represented an advanced recreational climber collective. Sporting or occupational stress on the fingers was considered an exclusion criterion for the control group. Participants were free to select the hand to be measured. None of the individuals had a contraindication for a 7 T high field MRI. All subjects gave written consent to participate and undergo the MRI examination, as well as to the use of their anonymized data. The study was approved by the institutional review board (260_15 Bc) and all patients provided informed consent. The study followed the declaration of Helsinki.

### Imaging

All imaging was performed on a 7 Tesla MRI scanner (MAGNETOM Terra, Siemens Healthineers, Erlangen, Germany). The subjects were examined in the superman position. The hand was fixed to reduce motion artifacts. A custom-made dedicated 1-channel transmit, 16-channel receive wrist coil (7 Tesla wrist coil, RAPID Biomedical, Würzburg, Germany) with an elliptical cross-section (78 mm × 98 mm) and a length of 70 mm was used. For each subject/specimen, T2-weighted multi-echo, spin-echo sequences (MESE) were acquired in the sagittal plane. T1-, T2-, and Proton density-weighted sequences in the axial direction, as well as three-dimensional double-echo steady state (DESS) sequences were obtained to visualize the finger anatomy and morphological joint changes. The latter served for subjective anatomical correlation and had no influence on the quantitative image analysis/region-of-interest (ROI) measurements. Details on the applied scanning parameters can be taken from Table 1.

**Table 1:** Details on the applied scanning parameters for 5 MRI sequences.

Parameter	T2 MESE	T1 TSE	T2 TSE	PD TSE	DESS
TR (msec)	3,030.0	700	68	14	17.97
TE (msec)	14.7–88.2	17	5,000	6,540	6.38
TA (min, sec)	8.31	4.46	6.32	6.32	8.55
Field of view	608 × 608	500 × 500	500 × 500	500 × 500	640 × 540
Flip angle	180°	90°	177°	177°	21°
Voxel size (mm)	0.2 × 0.2 × 2.5	0.2 × 0.2 × 1.5	0.2 × 0.2 × 1.5	0.2 × 0.2 × 1.5	0.3 × 0.3 × 0.3

### Image analysis/quantitative T2 mapping

**Figure 1:**
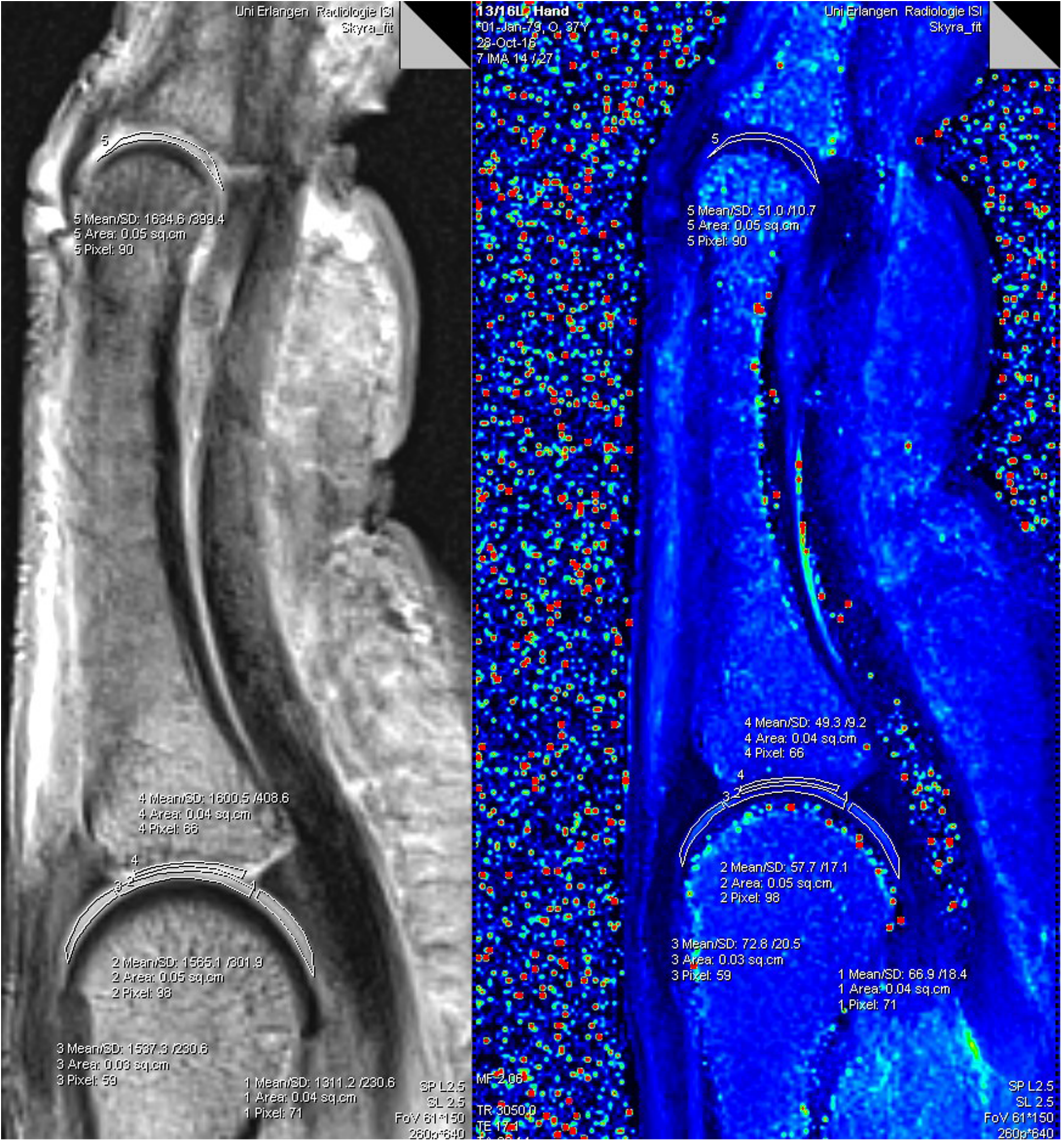
Software analysis for T2-mapping of a finger: the freehand drawn ROIs 1−5 in the morphological (grey left image) was transferred to the colour coded T2 map (right image). ROIs in the T2 map provide values for T2 time (Mean), standard deviation (SD), size of the individual ROIs (Area), as well as the number of pixels (Pixel).

All data sets were evaluated by two researchers in consensus regarding anatomical abnormalities/pathologies (M.B., 3 years; T.B.; 17 years of experience in musculoskeletal MRI). A ROI-analysis was performed with dedicated Software on a DICOM viewer (Leonardo syngo Multimo-dality Workplace VE36A; MapIt Software, both Siemens Healthineers, Erlangen, Germany). All ROIs were placed by the same researcher (M.B.) in consensus with (T.B.). A freehand drawing tool was used to create ROIs manually on the index, middle and ring finger of each individual in the central sagittal finger slice, which suited for optimal depiction of the metacarpophalangeal (MCP) and proximal interphalangeal (PIP) joint cartilage. The thicker cartilage layers of the MCP joint allowed an individual ROI placement at the proximal and distal joint side with three different cartilage segments defined for the MC head and with one cartilage segment defined for the ground phalanx base. The thinner PIP joint cartilage layers allowed a common ROI placement including the ground phalanx head and middle phalanx base cartilage. The ROIs were defined as following: proximal MCP joint dorsal (ROI 1), proximal MCP joint central (ROI 2), proximal MCP joint palmar (ROI 3), distal entire MCP joint cartilage layer (ROI 4) and PIP joint including the entire proximal and distal cartilage layer (ROI 5). The MapIt software enabled fully automated parametric inline T2 mapping of the imaged cartilage. The results of each ROI were the mean values of the T2 relaxation times in milliseconds (msec), the standard deviation, the size of the ROI in square centimetres (sq.cm), as well as the number of pixels. A pixel-wise, monoexponential, non-negative least squares (NNLS) suitability analysis was used for image fusion of the resulting maps with the corresponding anatomic image (Figure 1).

### Statistical analyses

Data collection was performed with anonymization in Microsoft Excel. The statistical software program R (version 3.5.3., R Foundation for statistical Computing, Vienna, Austria) was used for calculation. Mixed modelling was performed to determine differences of T2 relaxation times in between the different ROI groups depending on age and climbing activity. Also, differences for gender and different joints (PIP joints vs. DIP joints) were tested. The median age was determined as a cut point for defining an additional group analysis depending on the individuals' age (≤26.5 y; >26.5 y) dividing the study collective and older and younger individuals. Unless stated otherwise, data were expressed as mean ± standard deviation (range). *P*-values <0.05 were considered statistically significant.

## Results

**Figure 2:**
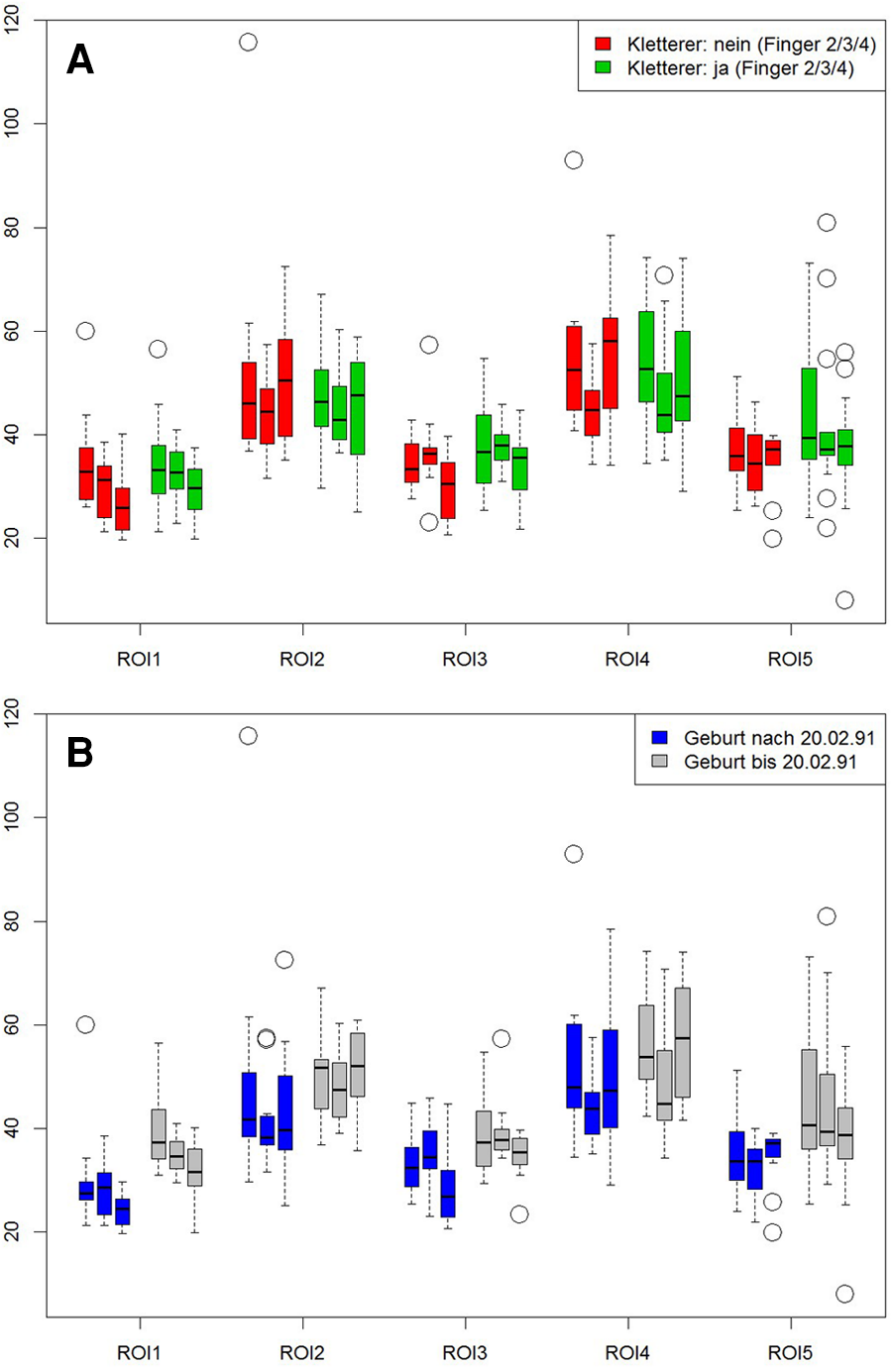
Influence of climbing and age on cartilage. ROIs 1 to 4 represent the metacarpal-phalangeal joints, ROI 5 reflects the proximal interphalangeal joint. Left bar = index, middle bar = middle finger, right bar = ring finger. First graph (**A**) shows the comparison between climbers (red bars) vs. non climbers (green bars). Second graph (**B**) compares subjects older (gray bars) vs. those younger (blue bars) 26.5 years of age.

MRI showed normal finger joint anatomy for all individuals without presence of any pathological or degenerative joint alteration and without presence of any osteophytes. T2-mapping was technically successful in all individuals and a total of 345 ROIs could be created with color-coded maps for subsequent ROI analysis. Sixteen subjects had imaging of the right hand and seven subjects of the left. The mean size of the ROIs was 0.04 cm^2^ and ranged from 0.01 cm^2^ to 0.13 cm^2^. Mean T2 values for all ROIs were 42.2 ± 7.78 msec for climbers and 41.4 ± 6.78 msec for non-climbers. The respective average T2 value was 44.2 ± 9.48 msec for older and 32.9 ± 5.71 msec for younger individuals. Further information on mean values of the T2 times of the individual ROIs, as well as their standard deviations (SD) are shown in Table 2 and Figure 2. Statistical analysis revealed no significant differences for T2 values between climbers and non-climbers. Within the MCP joints of all fingers, significant differences (*p* = 0.001) were found for the different ROIs 1,2,3 and 4. The averaged T2 time of all T2 times representing the MCP joint (ROI: 1–4) had no significant difference compared to ROI 5, representing the PIP joint. No significant difference was found between different genders. However, higher age had a significant impact on T2 values for all assessed ROIs (*p* = 0.001).

**Table 2:** Mean T2 values (ms) ± standard deviations of the entire study collective (*n* = 23), climbers (*n* = 13), non-climbers (*n* = 10), individuals ≤ 26.5 y (*n* = 13) and individuals > 26.5 y (*n* = 12) measured for three fingers and 5 different regions of interest (ROI) in-7 T MRI.

All participitants	ROI 1	ROI 2	ROI 3	ROI 4	ROI 5
Dig. 2	35.04 ± 12.75	49.84 ± 17.56	35.89 ± 7.04	54.74 ± 16.47	40.33 ± 13.77
Dig. 3	31.68 ± 10.78	44.85 ± 12.78	37.57 ± 7.93	46.47 ± 10.87	38.98 ± 11.33
Dig. 4	28.19 ± 8.86	47.63 ± 15.54	31.82 ± 9.72	53.8 ± 13.39	36.14 ± 10.57
Climbers					
Dig. 2	34.85 ± 9.51	55.56 ± 31.61	37.01 ± 8.24	59.15 ± 14.48	43.06 ± 14.49
Dig. 3	33.13 ± 5.01	44.92 ± 8.47	38.01 ± 4.45	47.74 ± 10.93	42.39 ± 16.6
Dig. 4	29.43 ± 5.58	54.64 ± 21.81	33.42 ± 6.47	52.22 ± 14.25	37.34 ± 11.99
Non-Climbers					
Dig. 2	46.48 ± 44.44	51.03 ± 17.85	34.42 ± 4.99	59.8 ± 28.69	36.77 ± 7.01
Dig. 3	29.15 ± 6.12	44.76 ± 8.47	37 ± 8.63	44.83 ± 7.77	34.54 ± 6.31
Dig. 4	26.58 ± 6.19	54.64 ± 21.81	29.75 ± 6.58	55.85 ± 13.08	34.59 ± 6.66
≤26.5 y					
Dig. 2	41.35 ± 41.38	48.52 ± 17.53	34.21 ± 5.36	55.68 ± 27.35	34.92 ± 7.66
Dig. 3	29.08 ± 5.74	42.93 ± 8.23	37.61 ± 8.73	44.18 ± 6.56	32.28 ± 5.27
Dig. 4	24.9 ± 3.62	48.83 ± 22.17	28.26 ± 5.55	50.82 ± 14.52	33.43 ± 6.32
>26.5 y					
Dig. 2	38.34 ± 8.56	59.12 ± 33.09	37.72 ± 8.3	63.53 ± 19.83	46.23 ± 13.43
Dig. 3	34.52 ± 4.1	46.95 ± 8.08	37.53 ± 2.75	48.98 ± 11.48	46.28 ± 16.08
Dig. 4	31.78 ± 5.91	49.84 ± 7.99	33.82 ± 5.56	56.83 ± 12.29	39.11 ± 12.42

## Discussion

Our study used 7 Tesla MRI T2 mapping techniques to detect potential early cartilage changes in the fingers in a collective of recreational young climbers. In comparison with an age-matched group of non-climbers, no differences in cartilage quality was found. Independent of the climbing sport, we found significant differences in cartilage composition in regard to age. We were able to define five distinct cartilage regions of the MCP and PIP joints of each finger, indicating feasibility of this T2 mapping technique for evaluating finger joint cartilage composition with MRI.

Several studies have investigated joint tissue adaptions in finger joints of climbers. Pastor et al. reported that the thickness of the cartilage layer in the PIP and DIP joints will decrease whereas the occurrence of osteophytes will increase during the career of elite sport climbers (12, 13). However, a clear connection between changes in the thickness of the cartilage, the development of osteophytes and degenerative symptoms could not be shown in their 10-year follow-up study (12, 13). In this context, it was discussed that osteophyte development in young climbers could be a mechanical adaptation to finger-stressing, which does not necessarily have to be accompanied by cartilage degeneration (12, 13). Schöffl et al. reported that one quarter of the German youth national team climbers showed a mild form of osteoarthritis Grade 2, however this study based on radiographic and clinical evaluation only (14). With integration of MRI assessment, a more comprehensive assessment on the cartilage quality may be possible in comparison to clinical, ultrasonographic or radiographic evaluation alone. Compositional MRI is suited to detect possible early pre-morphologic cartilage changes with T2 realxometry being the most widely applied technique as a surrogate parameter of collagen content and organization and water content of cartilage (15). Compositional imaging with finger T2 mapping was applied clinically in a study by Renner et al. (21), who investigated cartilage of MCP joints of patients with underlying rheumatic disease. In their study, inflammatory activity correlated to changes of the cartilage T2 times.

Our methodology differs to the aforementioned study, as a compositional imaging technique was used with the highest clinically approved magnetic field strength of 7 Tesla which allowed for maximized spatial resolution and an increased signal to noise ratio in comparison to 3.0 Tesla. Therefore, we could perform T2 mapping analysis also for the smaller PIP joint.

The mechanisms leading to cartilage degradation due to repetitive strain and/or aging are assumed to contribute to a decrease in proteoglycans and to changes of the collagen network. This subsequently yields to an increase of cartilage permeability and water content as the earliest demonstrable changes in cartilage damage (22), typically localized and quantified by the T2 relaxation time. In our study, a gender-specific difference in T2 times could not be shown. We found differences in T2 times for the different finger and ROI groups, which did not show a statistically significance, in relation to climbing sport and age using mixed modelling. In this respect, differing T2 values for the different ROI groups of the MCP may be interpreted as physiologic normal observation. It is possible that with future technical improvements (e.g., dedicated 7.0 Tesla hand/finger receiving MRI coil), further knowledge about that may be possible in subsequent studies using a more precise segmentation of the distinct cartilage layer segments also for the PIP joint and DIP joint (analogous to MCP).

7 Tesla MRI is not commonly applied clinically, although regulatory approval for routine clinical use has been granted. The advantages of a decreased acquisition time and higher spatial resolution at high magnetic field strength is advantageous for visualization of small anatomical structures such as the wrist (23) or finger joints (24).

Manual segmentation of the cartilage is dependent from the subjective judgement of each observer, which means that measurement accuracy can vary both between different observers and with repeated assessment by the same observer. In addition, manual T2 mapping takes a lot of time. Simplification could be achieved by developing automated assessment or evaluation tools with artificial intelligence algorithms, not only for 7 Tesla but also for the more widespread 3.0 Tesla installations, which would be easily accessible for follow-up studies on larger collectives. However, to date manual segmentation is still considered the gold standard technique (17).

Regarding the prevention of cartilage degeneration in climbers, comprehensive MRI analyses such as in our study could help to better understand the different stress mechanisms to finger cartilage segments. The influence of grip techniques such as slope grip or crimp grip (25, 26) on the cartilage at different cartilage locations could be investigated with compositional MRI techniques as in our study. Likewise, differences between diverse climbing disciplines (sport climbing, multi-pitch, bouldering, speed climbing) or different forms of training (static training, dynamic training) would be accessible in future studies. It is not yet understood whether climbing related bony adaptations, such as osteophytes or cortical thickening, are associated with cartilaginous damage in older athletes, or whether they are merely mechanical reactions as discussed before (13). Further knowledge on that will be increasingly more important with the gaining popularity of climbing.

Our study has several limitations. It is a small cross-sectional study and therefore does not allow any conclusions to be drawn about the changes found in the T2 times with regard to the actual occurrence of OA. We did not correlate MRIs with ultrasound/radiography and no analysis of the cartilage thickness was performed. An analysis for the DIP joint was not possible due to technical limitations, particularly owing to the coil design. The MRIs had to be performed without the use of dedicated hand/finger receiving coils, and as such are not yet commercially available. Further experience with larger collectives and with corresponding clinical classification into different degrees of osteoarthritis is necessary, in particular to better assess the sensitivity of the method to changes in cartilage in climbers. Such studies also could evaluate a possible reversibility of T2 time changes through adaption of climbing techniques and/or training methods, especially with consideration of clinical symptoms.

At this stage, the current data support the concept that the assessment of compositional T2 relaxation times reflects non-specific changes in cartilage in terms of early sport and age dependent alteration. Our study showed that T2 mapping is a feasible method for the direct evaluation of cartilage composition in young climbers. In our limited study collective, we did not register early onset cartilage changes dependent to climbing sport activity, whereas age-related effects seemed to be more prominent. T2 mapping seems appropriate as methodology to depict early hydration changes and collagen fibre damage in finger joint cartilage of larger subsequent studies.

## Data Availability

The original contributions presented in the study are included in the article/Supplementary Material, further inquiries can be directed to the corresponding author.

## References

[1] Lutter C El-Sheikh Y Schoffl I Schoffl V. Sport climbing: medical considerations for this new Olympic discipline. Br J Sports Med. (2017) 51(1):2–3. 10.1136/bjsports-2016-096871 27821387

[2] Lutter C Tischer T Schoffl VR. Olympic Competition climbing: the beginning of a new era-a narrative review. Br J Sports Med. (2021) 55(15):857–64. 10.1136/bjsports-2020-102035 33036996

[3] Cole KP Uhl RL Rosenbaum AJ. Comprehensive review of rock climbing injuries. J Am Acad Orthop Surg. (2020) 28(12):e501–9. 10.5435/JAAOS-D-19-00575 32015250

[4] Rooks MD. Rock climbing injuries. Sports Med. (1997) 23(4):261–70. 10.2165/00007256-199723040-00005 9160482

[5] Schoffl V Simon M Lutter C. Finger and shoulder injuries in rock climbing. Orthopade. (2019) 48(12):1005–12. 10.1007/s00132-019-03825-3 31705177

[6] Schöffl V Winkelmann HP. Injury-risk on indoor climbing walls. Ö J Sportmed. (1999) 3:53. 10.1055/s-2007-993308 10407959

[7] Woollings KY McKay CD Emery CA. Risk factors for injury in sport climbing and bouldering: a systematic review of the literature. Br J Sports Med. (2015) 49(17):1094–9. 10.1136/bjsports-2014-094372 26009554

[8] Rauch S Wallner B Strohle M Dal Cappello T Brodmann Maeder M. Climbing accidents-prospective data analysis from the international alpine trauma registry and systematic review of the literature. Int J Environ Res Public Health. (2019) 17(1). 10.3390/ijerph17010203 PMC6981967 31892182

[9] Miro PH van Sonnenberg E Sabb DM Schoffl V. Finger flexor pulley injuries in rock climbers. Wilderness Environ Med. (2021) 32(2):247–58. 10.1016/j.wem.2021.01.011 33966972

[10] Allenspach P Saupe N Rufibach K Schweizer A. Radiological changes and signs of osteoarthritis in the fingers of male performance sport climbers. J Sports Med Phys Fitness. (2011) 51(3):497–505. 21904290

[11] Frohlich S Schweizer A Reissner L Pastor T Sporri J Pastor T. Long term evolution of soft tissue response in the fingers of high-level sport climbers: a cross-sectional 10 year follow-up study. Phys Ther Sport. (2021) 52:173–9. 10.1016/j.ptsp.2021.09.006 34547601

[12] Pastor T Frohlich S Sporri J Schreiber T Schweizer A. Cartilage abnormalities and osteophytes in the fingers of elite sport climbers: an ultrasonography-based cross-sectional study. Eur J Sport Sci. (2020) 20(2):269–76. 10.1080/17461391.2019.1631389 31184978

[13] Pastor T Schweizer A Reissner L Pastor T Sporri J Frohlich S. Long-term evolution of cartilage abnormalities and osteophytes in the fingers of elite sport climbers: a cross-sectional 10-year follow-up study. Eur J Sport Sci. (2022) 22(9):1452–8. 10.1080/17461391.2021.1943716 34121624

[14] Schöffl VR Schoffl I. Finger pain in rock climbers: reaching the right differential diagnosis and therapy. J Sports Med Phys Fitness. (2007) 47(1):70–8. 17369801

[15] Heiss R Janka R Uder M Nagel AM Trattnig S Roemer FW. Update cartilage imaging of the small joints: focus on high-field MRI. Radiologe. (2019) 59(8):732–41. 10.1007/s00117-019-0521-x 30953080

[16] Crema MD Roemer FW Marra MD Burstein D Gold GE Eckstein F Articular cartilage in the knee: current MR imaging techniques and applications in clinical practice and research. Radiographics. (2011) 31(1):37–61. 10.1148/rg.311105084 21257932 PMC6939857

[17] Zibetti MVW Menon RG de Moura HL Zhang X Kijowski R Regatte RR. Updates on compositional MRI mapping of the cartilage: emerging techniques and applications. J Magn Reson Imaging. (2023) 58(1):44–60. 10.1002/jmri.28689 37010113 PMC10323700

[18] Springer E Bohndorf K Juras V Szomolanyi P Zbyn S Schreiner MM Comparison of routine knee magnetic resonance imaging at 3 T and 7 T. Invest Radiol. (2017) 52(1):42–54. 10.1097/RLI.0000000000000303 27434621

[19] Zilkens C Tiderius CJ Krauspe R Bittersohl B. Current knowledge and importance of dGEMRIC techniques in diagnosis of hip joint diseases. Skeletal Radiol. (2015) 44(8):1073–83. 10.1007/s00256-015-2135-3 25913097

[20] Ladd ME Bachert P Meyerspeer M Moser E Nagel AM Norris DG Pros and cons of ultra-high-field MRI/MRS for human application. Prog Nucl Magn Reson Spectrosc. (2018) 109:1–50. 10.1016/j.pnmrs.2018.06.001 30527132

[21] Renner N Kleyer A Kronke G Simon D Sollner S Rech J T2 mapping as a new method for quantitative assessment of cartilage damage in rheumatoid arthritis. J Rheumatol. (2020) 47(6):820–5. 10.3899/jrheum.180728 31416926

[22] Lazovic-Stojkovic J Mosher TJ Smith HE Yang QX Dardzinski BJ Smith MB. Interphalangeal joint cartilage: high-spatial-resolution in vivo MR T2 mapping–a feasibility study. Radiology. (2004) 233(1):292–6. 10.1148/radiol.2331031791 15317947

[23] Heiss R Weber MA Balbach E Schmitt R Rehnitz C Laqmani A Clinical application of ultrahigh-field-strength wrist MRI: a multireader 3-T and 7-T comparison study. Radiology. (2023) 307(2):e220753. 10.1148/radiol.220753 36625744

[24] Heiss R Librimir A Lutter C Janka R Kuerten S Roemer FW MRI Of finger pulleys at 7T-direct characterization of pulley ruptures in an ex vivo model. Diagnostics (Basel). (2021) 11(7). 10.3390/diagnostics11071206 PMC8303165 34359289

[25] Schweizer A. Biomechanical properties of the crimp grip position in rock climbers. J Biomech. (2001) 34(2):217–23. 10.1016/S0021-9290(00)00184-6 11165286

[26] Schweizer A Hudek R. Kinetics of crimp and slope grip in rock climbing. J Appl Biomech. (2011) 27(2):116–21. 10.1123/jab.27.2.116 21576719

